# Scalable Self-Powered Sensor Based on Triboelectric Nanogenerators with Surface-Modulated Electronegativity for Harsh Environments

**DOI:** 10.1021/acsami.5c12398

**Published:** 2025-09-25

**Authors:** Chen-Si Wu, Sz-Nian Lai, Ying-Hao Chu

**Affiliations:** † The Affiliated Chu Pei Senior High School of National Yang Ming Chiao Tung University, Hsinchu County 302, Taiwan; ‡ Department of Materials Science and Engineering, 34881 National Tsing Hua University, Hsinchu 300, Taiwan

**Keywords:** triboelectric nanogenerators, functionalizing process, self-powered sensors, electronegativity modulation

## Abstract

The growing need for self-powered sensors in extreme environments, such as biomedical implants, industrial monitoring, and deep-sea exploration, has driven interest in triboelectric nanogenerators (TENGs) as efficient energy harvesters. However, the challenge lies in developing a scalable, cost-effective fabrication process that maintains stable performance in water and across a range of varying temperatures. This study presents a surface modification strategy that enables precise modulation of electronegativity through a scalable and straightforward immersion process. Unlike conventional methods that rely on nanostructuring to enhance triboelectric activity, our approach utilizes surface functionalization to chemically anchor elements with varying electronegativities onto the substrate. These strong chemical bonds effectively modify the substrate’s electronegativity, thereby enhancing the TENG’s electrical output on both sides. The process is scalable beyond A4 size, making it well-suited for roll-to-roll manufacturing. By functionalizing polydimethylsiloxane (PDMS) electrodes with fluorine (−F) and amino (−NH_2_) groups, we significantly increase the triboelectric potential difference, enhancing charge transfer efficiency. Experimental results demonstrate that the NH_2_/fluorinert-modified TENG achieves an output voltage of 2.25 V and a current of 40 nA—an output current 600 times greater than that of pristine PDMS/PDMS. Additionally, theoretical simulations confirm a 225-fold increase in triboelectric potential, demonstrating the fundamental impact of electronegativity modulation. The device exhibits stable performance across a temperature range of 25–100 °C, in underwater conditions, following surface functionalization after thermal annealing, and under prolonged mechanical stress. This work represents a major breakthrough in scalable TENG fabrication, bridging laboratory innovation with commercial application. The demonstrated large-area fabrication approach unlocks new possibilities for wearable electronics, industrial sensing, and energy-efficient IoT devices, making self-powered technology more practical and accessible.

## Graphical abstract

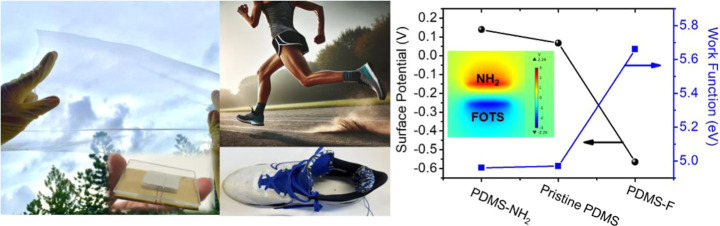

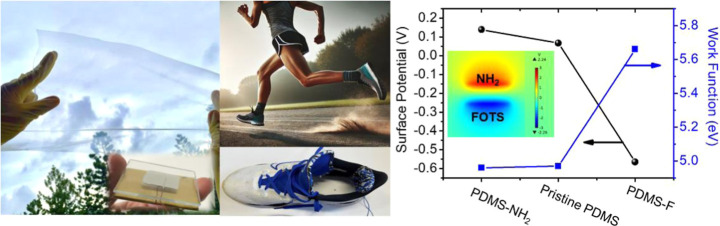

## Introduction

The global advancement of sensors has significantly influenced the internet of things (IoT), where interconnected devices rely on real-time data exchange for seamless operation.^1–5^ However, traditional sensors often depend on battery-powered systems with limited lifespans, posing sustainability challenges, particularly in environments where battery replacement is difficult or impractical. This limitation has driven a growing demand for self-powered sensors,^6–8^ especially in extreme conditions such as high-temperature and high-humidity regions, deep ocean environments, biomedical implants, and industrial monitoring systems.^9^ The triboelectric effect occurs when two materials with different electronegativities come into contact and then separate, resulting in charge transfer. The material with higher electronegativity attracts electrons from the other, generating an electric potential upon separation. This phenomenon is harnessed by triboelectric nanogenerators (TENGs) to convert mechanical energy into electrical energy efficiently.^10–13^ Their simplicity, cost-effectiveness, and compatibility with large-scale production make them ideal for sustainable sensing technologies that operate without external power.^14,15^ Due to these advantages, TENGs have demonstrated significant potential in energy harvesting,^16^ self-powered devices,^6,17,18^ and wearable electronics.^18^

Unlike conventional power generation methods, which often depend on complex and costly fabrication techniques, TENG devices offer the potential for low-cost production and easy integration into various platforms.^19,20^ However, despite their promising performance at the laboratory scale, a fully cost-effective and commercially viable process for large-scale fabrication (i.e., >A4 size) with optimized and stable performance has yet to be achieved. Maintaining consistent operation in water and across varying temperatures remains a key challenge. Overcoming these obstacles is critical to realizing the full potential of TENG technology in practical, commercial applications.

This study introduces a surface modification process for TENG fabrication that requires only a polyethylene terephthalate (PET) substrate, eliminating the need for complex synthesis and nanofabrication. Using a self-assembly approach, we precisely modulated surface electronegativity, maximizing the charge difference between electrodes through a scalable dipping process. Our goal is to develop a highly sensitive, stable, self-powered sensor while establishing a scalable manufacturing process suitable for roll-to-roll production. To enhance charge transfer efficiency, we utilize materials with distinct triboelectric potentials. Polydimethylsiloxane (PDMS) serves as a flexible,^21^ biocompatible material with naturally occurring hydroxyl (−OH) groups^22,23^ on its surface, facilitating effective charge interaction. As a consequence, the fluorine (−F)^24^ and amino (−NH_2_)^25^ groups were used to apply to both electrodes to maximize the triboelectric potential difference.

The amino group exhibits natural polarization, forming electron-rich sites capable of easily donating electrons to fluorinated surfaces during triboelectric interactions. Consequently, NH_2_-modified PDMS (NH_2_–PDMS) demonstrates enhanced charge generation, significantly increasing the electrical output of TENGs when paired with fluorine-functionalized PDMS as the opposing triboelectric layer. Our theoretical calculations confirm a remarkable 225-fold enhancement in triboelectric potential, highlighting the substantial effect of electronegativity modulation achieved with PDMS-NH_2_/PDMS-F compared to pristine PDMS. This TENG demonstrates promising potential for industrial and environmental monitoring, effectively detecting pressure variations in hazardous conditions and serving as a sustainable and reliable alternative to traditional sensing technologies.

## Results and Discussion

Figure 1a illustrates the concept of surface modification through an analogy of interlocking building blocks. This representation models the interaction between fluorinated molecules (fluoroctyltrichlorosilane, FOTS) and hydroxyl (−OH) groups on the PDMS substrate.^26^ In the inset of Figure 1a, the green block symbolizes surface hydroxyl groups, while the gray block denotes FOTS. Upon contact, these groups chemically bond to form a stable, modified surface layer. The lock-and-key analogy highlights the specificity and compatibility of the chemical interaction, facilitating improved adhesion and functional performance. As illustrated conceptually in Figure 1b, a PDMS layer is first deposited onto the PET substrate via spin coating, resulting in a PDMS/PET film stack. The following schematic outlines the chemical modification steps used to tailor the PDMS surface.^27^ These steps prepare the surface for the fabrication of the positive and negative triboelectric electrodes, as detailed below. First, the hydroxyl (−OH) groups naturally form on the PDMS surface in ambient air, creating a chemically active platform for further modifications.^28^ Two functionalization routes are shown: Route #1 uses FOTS to increase surface electronegativity, while Route #2 employs NH_2_ molecules (3-aminopropyltriehoxysilane, APTES) with NH_2_ groups to enhance positive charge affinity. For the negative triboelectric electrode in Route #1, when FOTS was formulated as an alcohol solution, its hydrolyzable end groups (Si–O–CH_3_) were converted into silanol groups (Si–OH) in FOTS. Consequently, the Si–OH groups condensed with hydroxyl groups (−OH) on the PDMS surface, forming covalent C–F bonds through a dehydration reaction that releases water (H_2_O). Accordingly, surface modification begins when FOTS (fluoroctyltrichlorosilane) reacts with the hydroxyl-rich PDMS surface. This process involves a dehydration (condensation) step, in which water molecules are removed as the silanol groups of hydrolyzed FOTS chemically bond with the hydroxyl (−OH) groups on the PDMS substrate, forming stable Si–O–Si bonds.^29^ The resulting covalent attachment ensures a permanent and stable modified surface layer that significantly enhances charge-transfer efficiency. Similarly, for Route #2 (Figure 1b), APTES undergoes the same condensation reaction with the substrate, forming stable Si–O–Si linkages while exposing terminal amine (−NH_2_) groups on the surface, which provide positively charged functional sites for improved performance as the positive triboelectric electrode. Figure 1c further illustrates that integrating the modified components into the TENG offers insight into the fabrication and operational mechanisms. The PDMS/PET film stack was chemically modified to create the positive and negative triboelectric electrodes, referred to as PDMS-NH_2_ (bottom electrode) and PDMS-F (top electrode), respectively. These two components were assembled with a polytetrafluoroethylene (PTFE) frame positioned between them, creating a gap for periodic contact and separation. The silver paste was then coated on both PET substrates with an external circuit attached for electrical data output, completing the process of self-powered sensor assembly.

**Figure 1:**
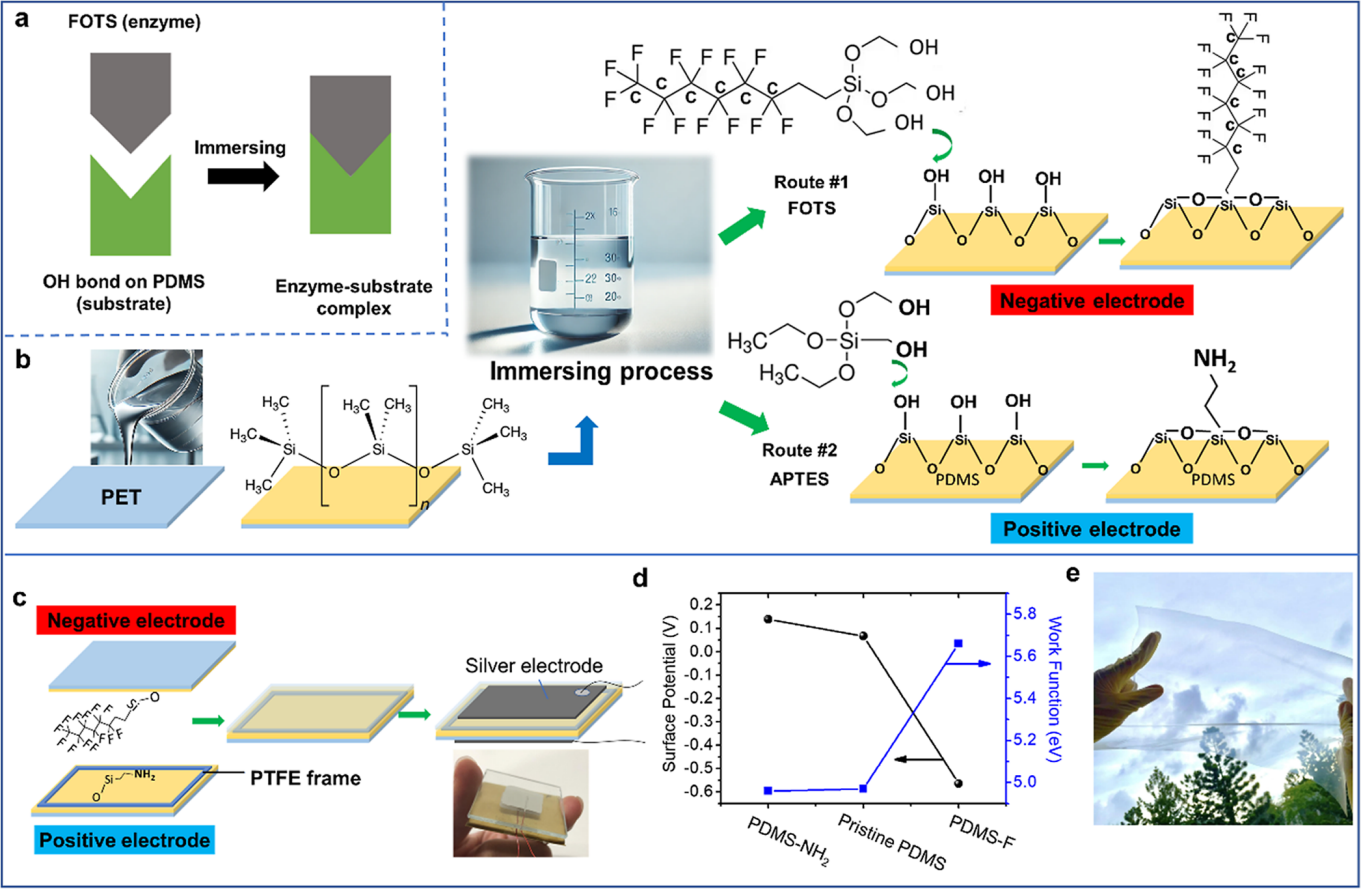
(a) Conceptual illustration of surface modification using FOTS and APTES through a simple immersion process. The schematic illustrates the chemical bonding of FOTS and APTES with surface hydroxyl groups (−OH) on PDMS, resulting in covalent Si–O–Si bonds. Route #1 modifies PDMS with fluorinated FOTS molecules to create the negative triboelectric electrode, while Route #2 uses amine-functionalized APTES to form the positive electrode. (b) Schematic of the fabrication process beginning with spin-coated PDMS on a PET substrate, followed by immersion in FOTS or APTES solution for functionalization. (c) Assembly of the triboelectric nanogenerator (TENG), comprising modified PDMS films as top (negative) and bottom (positive) electrodes separated by a PTFE spacer, with silver paste as the contact electrode. An inset shows the fabricated flexible device. (d) Work function and surface potential comparison of PDMS, PDMS-NH_2_, and PDMS-FOTS measured by Kelvin probe force microscopy (KPFM). The inverse trend between work function and surface potential highlights the effect of surface modification on triboelectric performance. (e) A4 size TENG.

Figure 1d presents a comparison of PDMS-NH_2_, pristine PDMS, and PDMS-F in terms of work function and surface potential, illustrating the impact of surface modification on the electronic properties of the material. The results reveal an inverse relationship between these two properties: as the work function increases, the surface potential decreases. This trend underscores the impact of surface modifications on charge transfer efficiency. In terms of work function, PDMS shows a slightly higher value than APTES-modified PDMS (PDMS-NH_2_), whereas FOTS-modified PDMS (PDMS-F) exhibits a significantly higher work function than both.^30^ Conversely, PDMS displays a slightly lower surface potential than PDMS-NH_2_, while both maintain substantially higher surface potentials compared to PDMS-F. This trend is explained by the electronegative nature of fluorine in FOTS, which lowers the surface potential by stabilizing electrons and reducing their escape energy. Meanwhile, the increase in work function for PDMS/FOTS is due to its enhanced ability to accumulate surface charge, a direct consequence of the strong electron-withdrawing effect of fluorinated groups. These findings align with the principles of the triboelectric effect, where materials with lower work functions tend to act as stronger electron donors, enhancing their triboelectric performance. The clear contrast between the stable work function and the varying surface potential confirms that while the material’s intrinsic properties remain unchanged, this emphasizes the importance of chemical functionalization in optimizing TENG performance, as it directly influences charge retention and energy generation efficiency. Additionally, the simple design enables the TENG to be fabricated at scalable sizes, extending up to at least A4 dimensions (Figure 1e).

Figure 2 shows X-ray photoelectron spectra (XPS) analysis of the triboelectric electrodes. The survey spectrum (Figure 2a) shows a distinct O 1s peak at ∼532.0 eV, which can be deconvoluted into two components at 532.9 and 532.2 eV, corresponding to Si–OH^26^ and Si–O–Si bonds, respectively. This indicates the presence of surface −OH groups on PDMS, which are strongly bonded within the polymer’s Si–O–Si network. The Si–OH groups on PDMS play a crucial role in facilitating the bonding of the end groups of FOTS and APTES to the PDMS surface. Figure 2b presents the F 1s spectrum of PDMS-FOTS. The carbon–fluorine (C–F) bonding exhibits a binding energy peak at approximately 689.6 eV, which can be attributed to the terminal fluorine group covalently bonded to the Si–O bonds on the PDMS surface. In Figure 2c, the N 1s spectrum of PDMS-APTES exhibits a distinct peak, similar to that observed in PDMS/PET. This peak can be deconvoluted into two components with binding energies of 401.6 and 399.7 eV, which are attributed to protonated amine groups (C–NH_3_^+^) and neutral amine groups (C–NH_2_), respectively. The presence of protonated amines suggests surface interactions and hydrogen bonding on the modified PDMS, while the neutral amines confirm the attachment of APTES. Figure 2d shows the F 1s spectrum for PDMS-FOTS after being heated at 80 °C for 2 h. The peak at 689.7 eV indicates the presence of stable C–F bonds, identical to the spectrum obtained after thermal treatment. Likewise, Figure 2e illustrates the N 1s spectrum for PDMS-APTES under the same heating conditions. The two characteristic peaks at 401.8 eV (protonated amines, C–NH_3_^+^) and 399.7 eV (neutral amines, C–NH_2_) are retained. These results confirm that the surface modifications using FOTS and APTES remain stable and effective even after exposure to elevated temperatures. The findings confirm that the surface modification is effective and scalable for high-throughput manufacturing.

**Figure 2:**
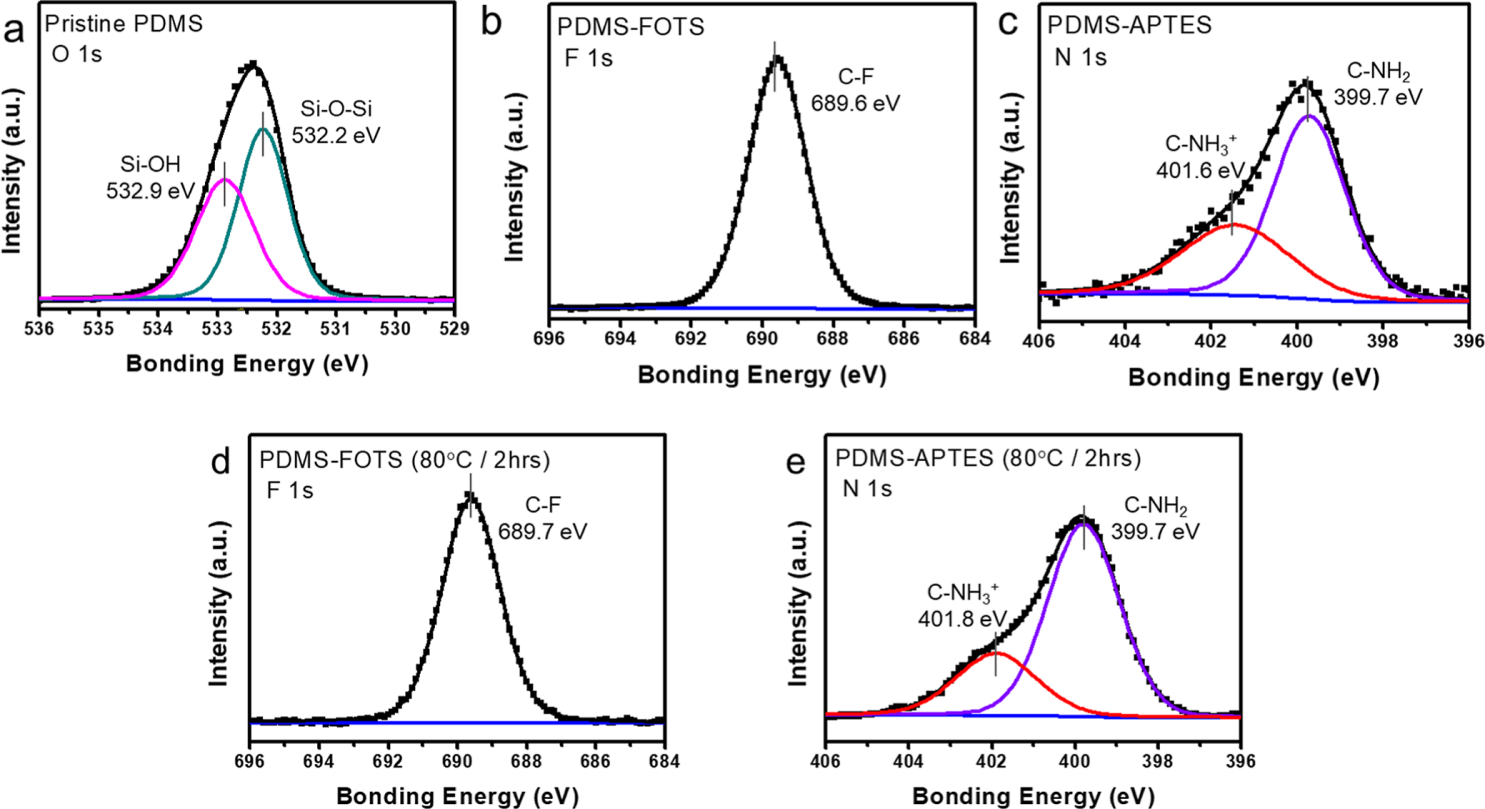
X-ray photoelectron spectroscopy (XPS) analysis of surface-modified PDMS electrodes. (a) O 1s spectrum of pristine PDMS, showing deconvoluted peaks at 532.9 eV (Si–OH) and 532.2 eV (Si–O–Si), indicating the presence of hydroxyl groups essential for surface functionalization. (b) F 1s spectrum of PDMS-FOTS displaying a distinct C–F peak at 689.6 eV, confirming the successful attachment of fluorinated terminal groups. (c) N 1s spectrum of PDMS-APTES showing peaks at 401.6 eV (protonated amines, C–NH_3_^+^) and 399.7 eV (neutral amines, C–NH_2_), indicating successful amine functionalization. (d) F 1s spectrum of PDMS-FOTS after thermal treatment at 80 °C for 2 h, showing a stable C–F peak at 689.7 eV, demonstrating thermal robustness. (e) N 1s spectrum of PDMS-APTES after the same thermal treatment, with retained peaks at 401.8 and 399.7 eV, confirming the stability of amine groups. These results verify the successful and thermally stable surface modification of PDMS with FOTS and APTES, enabling effective modulation of electronegativity for triboelectric applications.

Figure 3a–c illustrates the working mechanism and triboelectric interaction between PDMS-NH_2_ and PDMS-F in a contact–separation mode TENG. As shown in Figure 3a (steps I–IV), the TENG cycle begins with contact between the two surfaces (step I), allowing charge redistribution due to their differing electronegativities, and ultimately reaches a state of charge neutrality. Upon separation (step II), an electrostatic potential forms, driving electron flow through an external circuit. At the point of maximum separation (step III), the potential reaches its peak, generating the highest electrical output. As the layers return to contact (step IV), the charge imbalance is neutralized, completing the cycle and enabling continuous energy harvesting.

**Figure 3:**
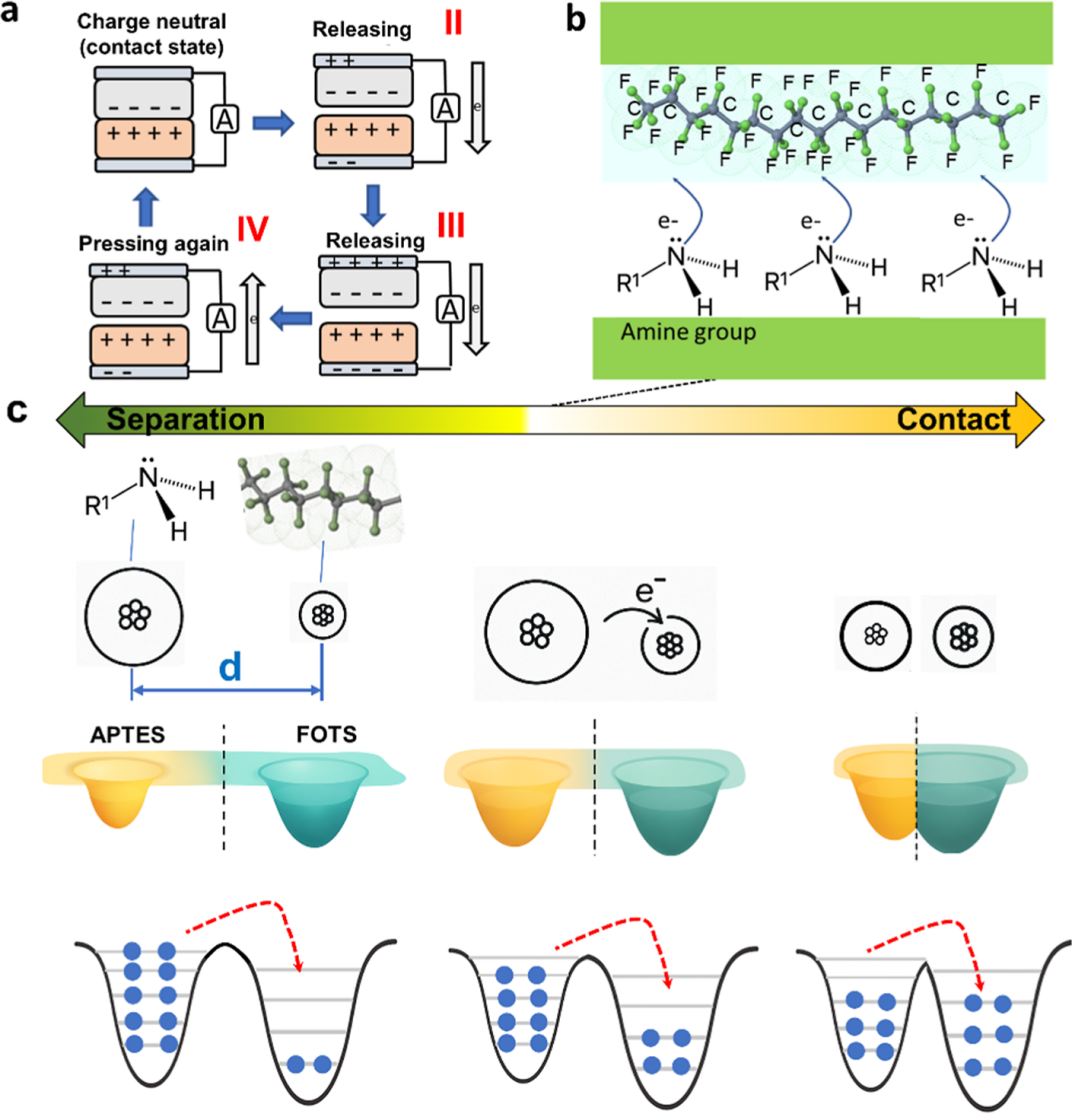
Triboelectric interaction mechanism and energy band alignment between PDMS-F (FOTS) and PDMS-NH_2_ (APTES). (a) When two materials—PDMS with amine groups (PDMS-NH_2_) and fluorinated PDMS (PDMS-F)—come into contact, electrons move from PDMS-NH_2_ to PDMS-F because the fluorinated surface strongly attracts electrons. When the surfaces pull apart, this charge difference creates an electric field, which pushes current through a connected circuit. This completes one cycle of energy generation. (b) Schematic of donor–acceptor roles during contact: PDMS-NH_2_ (donor) with lower effective work function transfers electrons to PDMS-F (acceptor) whose −CF_*x*_ dipoles raise the work function and stabilize acquired charge. (c) Band-diagram depiction of Fermi-level alignment at contact and charge retention after separation, illustrating electron flow from PDMS-NH_2_ to PDMS-F that underpins the device’s triboelectric output.

Charge-transfer mechanism for the NH_2_/fluorinated pair (Figure 3). As depicted in Figure 3b, amino-functionalized PDMS (PDMS-NH_2_, APTES-modified) behaves as the electron donor because of it is low electronegativity, whereas fluorinated PDMS (PDMS-F, FOTS-modified) acts as the electron acceptor during contact electrification due to it is high electronegativity. The lone pair on −NH_2_ creates electron-rich sites that lower the effective work function of PDMS-NH_2_, while the strongly electron-withdrawing fluorinated carbon groups on FOTS establish interfacial dipoles (Cδ^+^–Fδ^–^) that raise the work function of PDMS-F and stabilize acquired negative charge. This donor–acceptor assignment is consistent with our KPFM measurements in Figure 1d (PDMS-F shows a higher work function/surface potential relative to PDMS-NH_2_).

The resulting energy-level alignment is summarized in Figure 3c. Upon intimate contact, local orbital overlap and short-range tunneling drive electron flow from PDMS-NH_2_ to PDMS-F until interfacial Fermi levels equilibrate. On separation, electrons remain trapped on the fluorinated surface due to dipole-stabilized surface states, while residual positive charge persists on the amine-terminated surface; a fraction of surface amines becomes protonated (−NH_3_^+^), which we observe as characteristic N 1s features in XPS (Figure 2). The persistent C–F environment in XPS further supports robust negative-charge retention on PDMS-F. Together, Figures 3b–c, 1d, and 2 provide a coherent, measurement-anchored picture of electron-transfer-dominated contact electrification for this material pair.

This directional charge movement establishes the potential difference required for triboelectric generation and explains the enhanced device output under repeated contact–separation cycles. In brief, electron transfer from PDMS-NH_2_ to PDMS-F (contact), followed by charge trapping on PDMS-F (separation), yields larger net surface charge densities^31^ and thus higher voltage and current than pristine PDMS/PDMS, in agreement with prior reports on amine/fluorinated interfaces.^32–34^

Figure 4a,b present the output voltage and current, respectively, for pristine PDMS, PDMS/PDMS-F, and PDMS-NH_2_/PDMS-F TENGs. As shown in Figure 4a, the pristine PDMS TENG generated the lowest output, around 0.25 V, due to its minimal electronegativity contrast. When fluorinert was surface-modified on PDMS (PDMS-F), its increased electronegativity enhanced the electrical output to 0.5 V due to the greater electronegativity contrast with pristine PDMS. Moreover, the most significant voltage enhancement was observed with the PDMS-NH_2_/PDMS-F combination, which produced the highest output of 2.25 V, 9 times greater than that of pristine PDMS TENG. Similarly, Figure 4b shows that the PDMS-NH_2_/PDMS-F combination achieved the highest output current, reaching 40 nA, approximately 600 times higher than pristine PDMS. This improvement is attributed to the relatively strong positive charge of NH_2_ and the high electronegativity of fluorinert after surface modification, which maximizes triboelectric charge separation. In contrast, pristine PDMS exhibited only a minimal output current. These results highlight the crucial role of surface functionalization in optimizing TENG performance.^35^ Therefore, instead of merely selecting different materials, modifying the surface chemistry enhances electron transfer efficiency, significantly improving self-powered sensing performance. Polarity check of the TENG by swapping output leads (Figure S1, Supporting Information of S1), confirms that reversing the leads only inverts the sign of *I*_sc_ without changing the period or magnitude.

**Figure 4:**
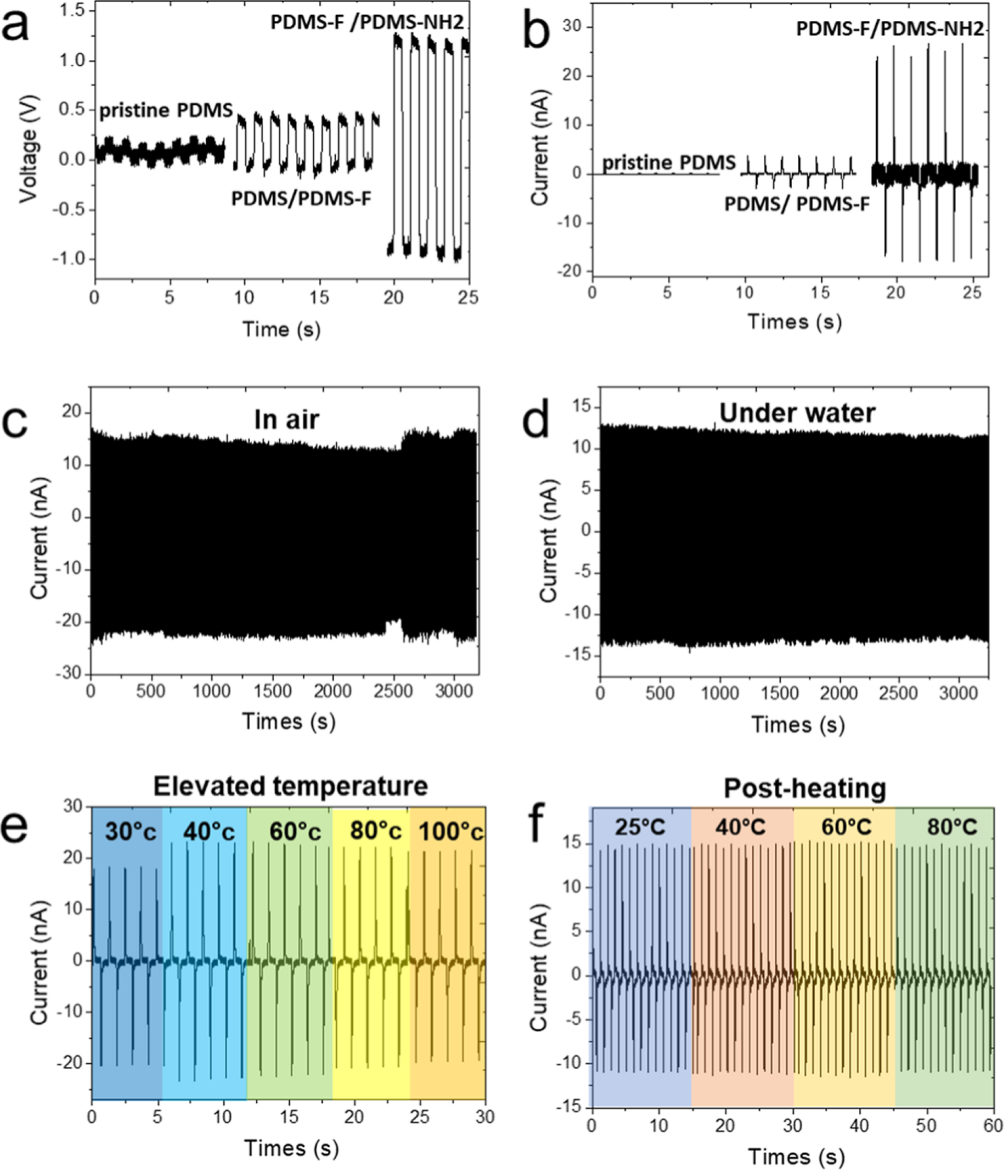
Electrical performance and environmental stability tests of triboelectric nanogenerators (TENGs) with different surface modifications. (a) Output voltage comparison among PDMS/PDMS, PDMS/FOTS, and PDMS-NH_2_/PDMS-F configurations, showing significantly higher voltage from the NH_2_/F functionalized TENG (up to 2.5 V). (b) Corresponding output current measurements, with PDMS-NH_2_/PDMS-F achieving the highest current output (∼40 nA), approximately 10 times greater than pristine PDMS. (c) Long-term durability test of the TENG current in air, showing stable operation over 3000 s. (d) Durability test under water, confirming continuous and consistent output in submerged conditions. (e) Current output performance at varying water temperatures (30 °C, 40 °C, 60 °C, 80 °C, and 100 °C), indicating thermal robustness across a broad temperature range. (f) Thermal annealing stability test at 25 °C, 40 °C, 60 °C, and 80 °C for 2 h each, demonstrating consistent output and practical functionality after prolonged thermal exposure.

To assess the durability of the as-prepared TENG, we selected the PDMS-NH_2_/PDMS-F combination to evaluate its electrical output under different environmental conditions, including air vs underwater long-period performance, elevated temperature, and postheating test. To assess the TENG’s long-term operational performance, tests were conducted continuously for 1 h in both air and underwater environments, as shown in Figure 4c,d, respectively. The comparison of electrical output in air versus underwater demonstrates stable performance in both conditions. Although the signal amplitude is slightly lower in air, the consistent waveform confirms reliable charge transfer and functionality. The approach is readily adaptable for industrial-scale manufacturing, improving the practicality and economic viability of TENG commercialization. (See Live Video #1, Supporting Information).

Figure 4e displays the TENG’s output at various water temperatures (30 °C, 40 °C, 60 °C, 80 °C, and 100 °C), with signal traces color-coded from blue to orange to represent increasing temperature (see Live Video #2 in Supporting Information). The consistent signal patterns across all temperature points indicate that temperature variations do not affect the sensor’s performance, demonstrating the TENG’s thermal reliability. To evaluate whether PDMS electrodes functionalized with fluorine (–F) and amino (−NH_2_) groups can withstand thermal annealing, the TENG was subjected to heating cycles at various temperatures. Figure 4f presents the electrical output after thermal exposure at 25 °C, 40 °C, 60 °C, and 80 °C for 2 h at each temperature. The results demonstrate stable and consistent output across the entire temperature range, characterized by uniform waveforms. These findings confirm that the sensor maintains reliable functionality and effective charge transfer even after repeated thermal cycling. Current density and power density vs external load resistance for 4 × 4 cm device (Figure S2, Supporting Information of S2). The device delivers a peak areal power density of ∼60 mW m^–2^ near ∼300–350 MΩ.

To evaluate its practicality, we installed the TENG on the soles of running shoes and conducted a running test to simulate real-world human motion. Figure 5a presents a TENG’s simulation of the foot-stepping test, modeling mechanical and electrical responses under pressure. By embedding the device in a sneaker for data collection, the analysis provides insights into contact behavior, stress distribution, and energy harvesting, highlighting its potential for motion-powered sensing electronics. The results demonstrated a significant charge transfer efficiency in the PDMS-NH_2_/PDMS-F configuration, generating a consistent electrical output across various physical activities, including jumping, jogging, and walking, as shown in Figure 5c. Note that the output voltage (Figure 5b) and current (Figure 5c) were measured at different times, resulting in different operating frequencies. The result shows that jumping’s output was the highest, followed by jogging and walking. The promising results from the foot-stepping test confirmed that the PDMS-NH_2_/PDMS-F TENG is well-suited for integration into interactive electronics. It is worth noting that, as this was a real-time test, the conductor’s body weight was directly applied to the TENG, exerting considerably higher pressure compared to the experiments shown in Figure 4a–f, which were conducted using a linear motor. As a result, the generated current was significantly increased relative to the general tests presented in Figure 4.

**Figure 5:**
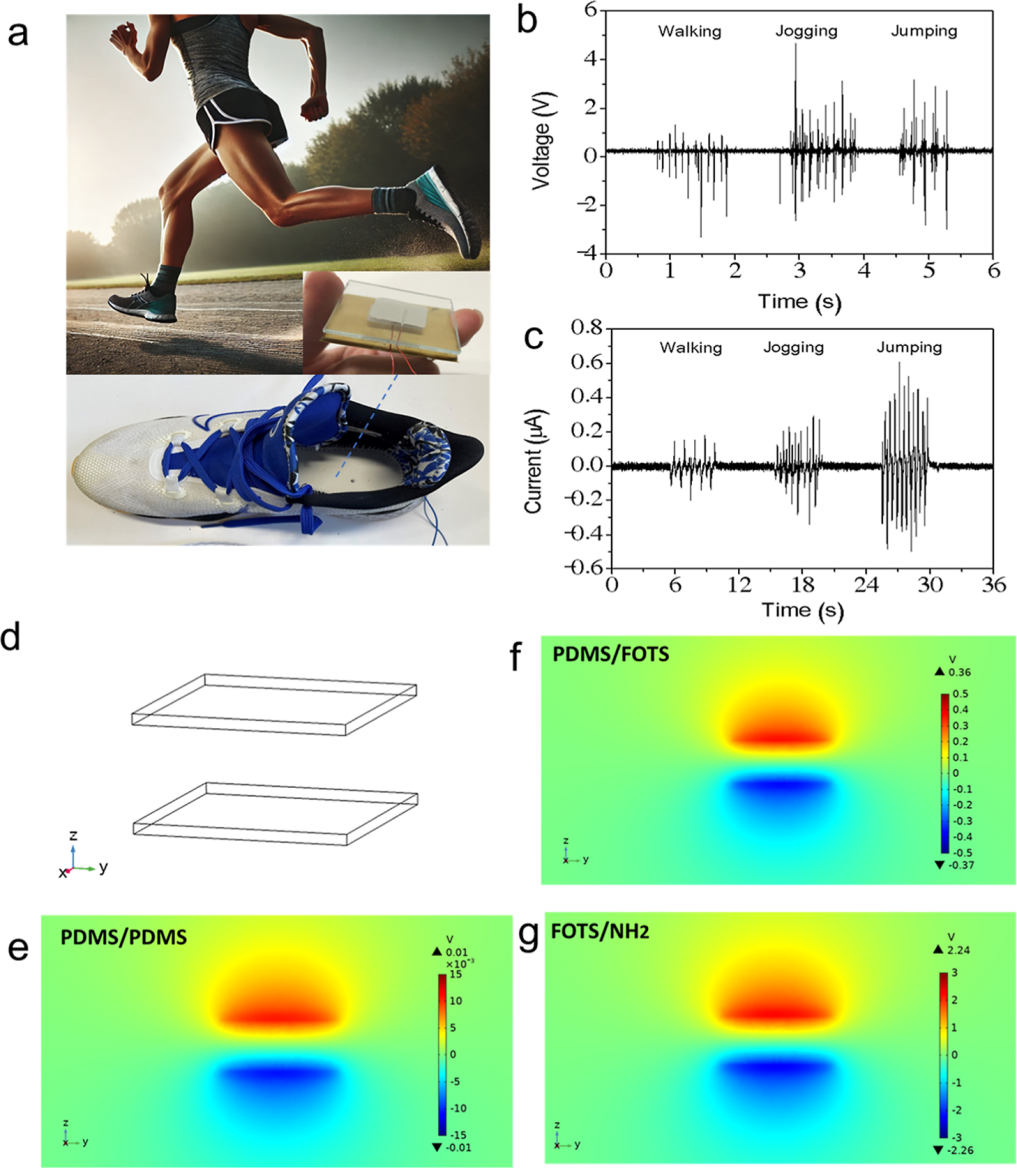
(a) Real-world application of the TENG device embedded in a running shoe to harvest biomechanical energy during foot motion. The inset shows the fabricated PDMS-NH_2_/PDMS-F device used for testing. (b) The corresponding output voltage of the TENG embedded in a running shoe under various real-life motion conditions. (c) The corresponding output current of the TENG embedded in a running shoe under various real-life motion conditions. (d) Simplified 3D model of the TENG structure used in COMSOL simulations, showing two opposing electrode layers. (e–g) Simulated electric potential distributions under contact-separation conditions for different material configurations: (e) PDMS/PDMS shows minimal potential generation due to low electronegativity contrast. (f) PDMS/PDMS-F exhibits enhanced potential (±0.36 V) due to fluorinated surface modification, which increases surface charge separation. (g) PDMS-F/PDMS-NH_2_ configuration exhibits the highest potential (∼± 2.24 V), confirming that the significant electronegativity difference between NH_2_ and FOTS optimizes triboelectric performance for energy harvesting applications.

The simulation calculations presented in Figure 5d analyze the electric potential distribution in different TENG configurations, further illustrating the structural setup of the simulations, where two parallel plates represent the components’ contact surfaces involved in the triboelectric effect. These surfaces periodically contact and separate, generating triboelectric charges that induce an electrical potential difference.^36^ Figure 5e displays the PDMS/PDMS configuration, where both contact surfaces are unmodified PDMS. Since both materials have similar triboelectric properties, minimal charge transfer occurs, resulting in a very weak triboelectric potential—only a small potential difference (∼± 0.01 V), which highlights its low efficiency in charge generation. In contrast, the PDMS/PDMS-F configuration (Figure 5f) exhibits a significantly stronger charge separation due to the fluorinated modification, which increases the surface’s electronegativity. The simulation reveals a red-blue potential distribution, indicating enhanced charge transfer, with a potential range of approximately ±0.36 V. Despite using the same color scale, the distinct voltage bar confirms that PDMS/PDMS-F achieves a much higher triboelectric potential than unmodified PDMS/PDMS. The PDMS-NH_2_ (Figure 5g) enhances this effect by coupling with FOTS to form PDMS-NH_2_/PDMS-F, combining the highly electronegative PDMS-F surface with the strongly electropositive NH_2_ modification. This combination maximizes the electronegativity contrast, resulting in the highest recorded triboelectric potential (±2.25 V), which is 225 times greater than that of unmodified PDMS/PDMS. These results provide final confirmation that surface modification plays a crucial role in optimizing TENG performance, as increasing the electronegativity contrast between two materials leads to greater triboelectric charge transfer and higher electrical output. The PDMS-NH_2_/PDMS-F combination, demonstrating the most potent charge induction, proves to be the most effective configuration for high-performance self-powered sensors. Reinforcing the effectiveness of surface functionalization in TENGs. This novel surface-functionalized TENG design achieves high performance without the need for complex nanostructuring or expensive fabrication steps. Its simplicity, scalability, and material versatility make it a practical candidate for real-world integration. While not necessarily intended for mass production, the demonstrated approach offers a low-barrier, adaptable solution for developing self-powered sensors, particularly in customized or application-specific scenarios.

## Conclusion

This study presents a significant advancement in triboelectric nanogenerator (TENG) technology through a scalable, low-cost surface modification strategy that precisely modulates material electronegativity. By functionalizing PDMS electrodes with fluorine (–F) and amino (−NH_2_) groups via a simple immersion process, without the need for complex nanostructure fabrication, we demonstrate a highly effective approach to boost charge transfer efficiency. Experimental results confirm that the NH_2_/F-modified TENG achieves an output voltage of 2.25 V (9× higher than pristine PDMS/PDMS) and a current output of 40 nA (600× improvement), underscoring its superior electrical performance. Importantly, the process is scalable beyond A4 size, enabling compatibility with roll-to-roll manufacturing and practical for industrial applications. The device maintains stable and consistent output not only in ambient air but also in water, including under a wide temperature range (30–100 °C), demonstrating excellent environmental robustness. Theoretical simulations further corroborate these findings, showing a 225-fold increase in triboelectric potential for the NH_2_/F configuration compared to unmodified PDMS surfaces, reinforcing the critical role of surface electronegativity tuning. Together, these results validate a novel TENG design that balances simplicity, durability, and high output, without relying on nanostructured surfaces. This breakthrough holds strong potential for self-powered sensing systems in diverse real-world environments, including wearable electronics, industrial monitoring, and underwater applications.

## Experimental Section

To modify the surface of the TENG, first, polydimethylsiloxane (PDMS, Biocando) elastomer and cross-linker were uniformly mixed in a 10:1 weight ratio (w/w), and then spin-coated onto a polyethylene terephthalate (PET, 3 cm × 3 cm) substrate, forming a PDMS/PET stack film. Next, in a 500 mL glass beaker, anhydrous ethanol (99.8%, Fidher chemical) and a modification solution, such as 1*H*,1*H*,2*H*,2*H*-perfluorooctyltriethoxysilane (FOTS, purity: 97%, Alfa Aesar), were sequentially added in a volume ratio of 95:5. The total volume was adjusted to 200 mL after mixing. After that, a magnetic stir bar was placed in the solution, and the mixture was stirred at 300 rpm using a magnetic stirrer at room temperature and atmospheric pressure for 10 min to ensure uniform mixing. Finally, the PDMS/PET stack film was immersed in the solution for 3 h at 60 °C. After modification, the treated substrate was carefully removed using stainless steel tweezers, rinsed with deionized water and 95% ethanol (by volume), and dried in an oven at 50 °C to remove residual liquid. For surface modification using (3-aminopropyl) triethoxysilane (APTES, purity: 98%, Alfa Aesar), the same procedure was applied, including for large-scale samples of A4 size. As a result, PDMS-F/PET and PDMS-NH_2_/PET substrates with modified electronegativity were successfully prepared. X-ray photoelectron spectroscopy (XPS PHI 5000 Versaprobe, ULVAC-PHI) was conducted to determine the chemical states of the functionalized PDMS. Kelvin probe force microscopy (KPFM, Bruker Dimension ICON) was performed using a tapping mode to analyze the surface potential and work function of the modified samples. The output current and voltage of the TENG sensors were measured using a low-noise current preamplifier (Stanford Research System model SR570), a low-noise voltage preamplifier (Stanford Research System model SR560), and a programmable electrometer (Keithley 6514). Additionally, theoretical simulations of the triboelectric potential were conducted in electrostatic mode using COMSOL Multiphysics software.^37^ The geometrical parameters of the length (*L*), width (*W*), and height (*H*) of both sides of the triboelectrods were set to be 4 × 4 × 0.5 cm. The Layer nomenclature: PDMS-F = FOTS-modified PDMS (fluorinated, negative); PDMS-NH_2_ = APTES-modified PDMS (amine, positive); PDMS (pristine) = unmodified PDMS.

## Supplementary Material







## Data Availability

All data generated in this study are included in this published article. Additional data sets used during the study are available from the corresponding author upon reasonable request.

## Notes

Supporting Information for live video associated with this article can be found in the online version (PDF)
(MP4)
(MP4)
