# Supplementary material for: Scalable Self-Powered Sensor Based on Triboelectric Nanogenerators with Surface-Modulated Electronegativity for Harsh Environments
Source: ACS Appl Mater Interfaces. 2025 Sep 25;17(40):56454–63. doi: 10.1021/acsami.5c12398 (PMC12516684; doi:10.1021/acsami.5c12398)
Supplement: Supplementary file 1 [file am5c12398_si_001.pdf]

Supporting Information

Scalable Self-Powered Sensor Based on Triboelectric Nanogenerators with Surface-Modulated Electronegativity for Harsh Environments

Authors: Chen-Si Wu[1], Sz-Nian Lai[2], and Ying-Hao Chu[2]*

[1]The Affiliated Chu Pei Senior High School of National Yang Ming Chiao Tung University, Hsinchu County 302, Taiwan

[2]Department of Materials Science and Engineering, National Tsing Hua University, Hsinchu 300, Taiwan.

*Corresponding Author : Ying-Hao Chu

Email: yhchu@mx.nthu.edu.tw

Supporting Information of S1

Polarity check of the TENG by swapping the output leads. Figure S1a is the original wiring: the short-circuit current (I_{sc}) shows periodic bipolar pulses with a slightly larger negative peak. Figure S1b: After swapping the leads, the trace inverts about zero while the period and absolute peak values remain essentially unchanged. This confirms that lead assignment changes only the sign; magnitude-based metrics and harvested power after rectification are unaffected. All traces were recorded under identical contact–separation conditions.

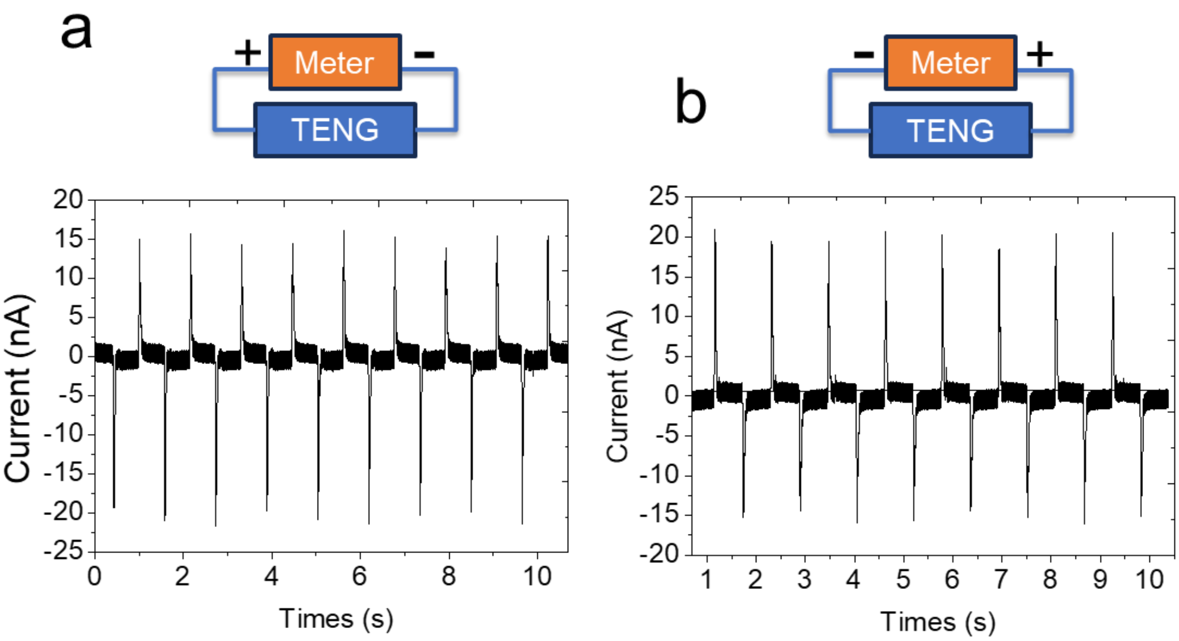

Figure S1. Switching polarity test (a) Original TENG sesor output (b) output after switching polarity

Supporting Information of S2

Current and power density as a function of external load resistance for a 4 × 4 cm device (Figure S2). Current density (black squares, left axis, μA m^{-2}) decreases with increasing resistance, while power density (blue squares, right axis, mW m^{-2}) rises across the measured range from ~0 Ω to 600MΩ. The maximum areal power density, ~60 mW m^{-2}, occurs at an external load of ~300–350 MΩ.

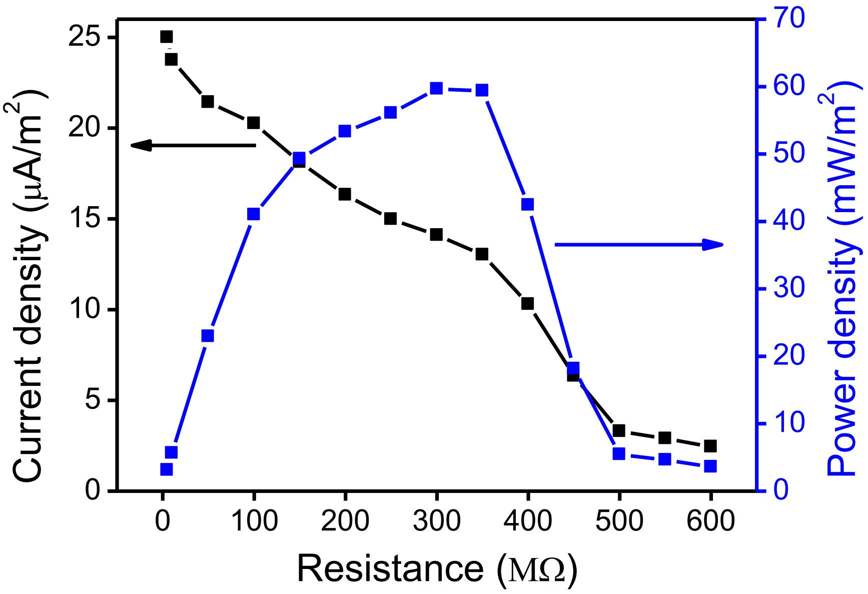

Figure S2. Power density and load data
